# A Review of Nutritional Guidelines and Menu Compositions for School Feeding Programs in 12 Countries

**DOI:** 10.3389/fpubh.2015.00148

**Published:** 2015-08-05

**Authors:** Ruzky Aliyar, Aulo Gelli, Salha Hadjivayanis Hamdani

**Affiliations:** ^1^Imperial College London, London, UK; ^2^Poverty, Health and Nutrition Division, International Food Policy Research Institute, Washington, DC, USA; ^3^Partnership for Child Development, Department of Infectious Disease Epidemiology, Imperial College London, London, UK

**Keywords:** school feeding, nutrition, guidelines, poverty, education

## Abstract

**Study objectives:**

To analyze the nutritional guidelines and menu compositions of school meal provision in various different countries.

**Background:**

School feeding is the provision of food on-site or to take home, which aims to increase school enrollment, attendance and retention, and exist as a social safety net for households with very low income. Home-grown school feeding, additionally, aims to stimulate local economies by providing a source of income for local smallholder farmers.

**Methods:**

Literature searches using the Ovid MEDLINE databases gathered information from in-country stakeholders and accessed the program websites of various countries. Nutrient composition of these menus was calculated from nutritional guidelines and menu compositions using a nutrition linear programing tool.

**Country comparisons:**

School feeding aims differ between countries of each income group. The implementation, delivery of service, and nutritional content of foods also differ considerably between countries and income groups. In high-income countries, guidelines and standards have been recommended in an attempt to combat rising levels of overweight and obesity, and to model healthier lifestyle habits. In low-income countries, there is a gap in terms of guidance on nutrition standards and menu composition.

**Conclusion:**

Provision of evidence-based guidance on nutrition standards to middle and low income countries, who have recently established or are planning to establish school feeding, has the potential to greatly enhance and improve the quality of service and improve the life of millions of children worldwide.

## Introduction

Nearly 805 million people in the world do not have enough to eat and 98% of them live in middle and low-income countries (1). Women make up over 60% of the hungry in the world while they represent just over 50% of the world population (2). High-income, and now increasingly middle- and low-income countries, have an additional social problem and challenge. Overweight and obesity levels are increasing and various studies have identified that schoolchildren consume unhealthy foods and lack adequate knowledge in healthy eating habits and lifestyle choices (3–6). School feeding is one important method of positively addressing these complex issues in all these countries.

### What is school feeding and home-grown school feeding?

School feeding (SF) is the provision of food on-site or to take home. Home-grown school feeding is a broad-based definition for SF programs where goods and services for meal preparation are procured from small-holder farmers and businesses. HGSF can be seen as a vehicle to stimulate local economies by providing a market and source of income for local smallholder farmers. In addition, it can also be used as a strategy to ensure that SF menus contain a variety of nutritious food that schoolchildren are accustomed to. These programs aim to achieve a variety of positive outcomes. The aims of school feeding differ according to country. This review focuses on school feeding in high-, middle-, and low-income countries. For each country, we have mentioned the main aims and objectives of the country-specific school feeding program (SFP), its framework, service delivery, nutritional guidelines (or the lack of it), and the nutritional composition of menus.

In high-income countries, SFPs aim to tackle the rising levels of childhood overweight and obesity. In middle- and low-income countries, SFPs have two different branches of aims. In the short-term, it aims to alleviate hunger, exist as a social safety net for households with very low income, and increase enrollment of children into schools (7). In the longer term, it aims to improve the nutritional status, attendance, cognitive development, and retention of school children (7). A Cochrane review, which contained trials from five continents and spanning eight decades, concluded that “school feeding programs significantly improve growth and cognitive performance of disadvantaged children” (8).

There are two main modalities of school feeding: in-school feeding and take-home rations (9). These are usually complemented with other interventions such as micronutrient supplementation, fortified biscuits, and deworming programs.

There is evidence that school feeding increases enrollment, attendance, retention, and educational achievement and alleviates short term hunger (10–12). However, further research must be conducted in order to determine how much of a significant longer-term benefit school feeding has over other social safety nets.

These positive aspects of school feeding are coupled with the negative aspects or trade-offs. There is plenty of evidence that show school feeding increases enrollment, attendance, and retention; however, this is not the case with the improvement of overall nutritional status of schoolchildren (10). It has been observed that in certain cases, SFPs have led to school children being fed less at home as some parents use the SFP as a replacement for feeding at home; whereas, in reality, it is meant to complement the child’s diet in addition to home-feeding (8). These families view the SFP as an income transfer and tend to spend the food budget of these schoolchildren on other household purchases. For a SFP to be successful, it must be ensured that this substitution effect does not take place.

Studies conducted in Ghana, Kenya, Mali, and Rwanda have concluded that there is a need for guidelines on nutrition and menu designs to be recommended in countries that have established HGSF programs (13). Currently, there are no nutritional guidelines in these countries and very little guidance on menu design.

This review analyses the nutritional guidelines and menu compositions of various countries in order to gauge the amount of nutrients that are being delivered through SFPs via on-site feeding, with a view to highlight where there is a need to establish and implement guidelines to improve the quality of life of schoolchildren. In England, France, USA, Italy, Finland, and Brazil, we focused mainly on the current nutritional guidelines and how national programs of school meal provision are implemented. In Ghana, India, Kenya, Mali, and Rwanda, we examined if a standard existed for SF programs, the nutritional content of menus, and how much local produce is incorporated into these menus.

## Methods

Relevant literature was searched using a variety of methods, including searches using the Ovid MEDLINE databases (from 1946 to present), interviews with program stakeholders in Nigeria, Kenya, and Ghana, and through school food program websites of various countries. We focused on on-site feeding and lunch time meals only. We used reviews to obtain nutritional guidelines, and studies and state school feeding documents to obtain menu compositions. We compared these nutritional values with the Recommended Daily Allowance (RDA) advised by the World Health Organization (WHO) for each nutrient. Nutritional guidelines were obtained for England, France, USA, and Brazil and the percentage composition of nutrients was calculated using these guidelines. There are no legislated or advised nutritional guidelines in Ghana, India, Kenya, Mali, and Rwanda: nutritional values were calculated using the “*NutVal*” nutritional value calculator, (12) from a sample of menu compositions found for each country in the literature. For Ghana, we were not able to find guidelines or the daily ration amount for the menu composition, so values were estimated in comparison with other countries in the region. Nutritional guidelines or menu compositions for Italy and Finland were not available. For countries without references to the cost per meal, the cost per daily meal per child was calculated using average costs of SFPs of these countries. All currency units were converted into US dollars using the online XE Currency Converter (14). Data were collected and compared for 12 countries classified into their respective income groups as per World Bank classifications (15).

## Nutrient Intake

Recommended nutrient intake (RNI) is the daily intake that meets the nutrient requirements of almost all apparently healthy individuals in an age and sex-specific population (Table 1). There may be differences in the equivalence for different countries. RNI is equivalent to that of recommended dietary allowance (RDA) as used by the Food and Nutrition Board of the US National Academy of Sciences. RNI are usually captured for both macronutrients and micronutrients. *Macronutrients* (basically carbohydrates, protein, and fat) provide the energy (kilocalories) needed by the body to maintain essential body functions, growth, and physical activities. The recommended level of dietary energy intake for a population group, which is the mean energy requirement of healthy, well-nourished individuals who constitute that group, may differ slightly depending on the situation. Children and adolescents in rural, traditional communities of developing countries, for example, are more active than their counterparts living in urban areas, or children from developed, industrialized countries, and hence, may have a slightly different requirement. The FAO/WHO/UNU Consultation (2004) endorsed the recommendation to reduce or increase by 15% of the requirement of population groups that are less or more active than average, starting at 6 years of age. (16)

**Table 1:** **Recommended ranges of nutrient intakes**.

Dietary component	Goal expressed as % of total energy
Total carbohydrates	55–75
Total fat	15–30
Proteins	10–15
Free sugars	<10

*Source: WHO/FAO 2004 (16)*.

There are important differences in energy and nutrient requirements between boys and girls. These differences increase with age, at approximately 5% for boys aged 6, and up to 15% for older boys at around 14 years. Because these differences are minor, there is no need to allocate different rations in mixed schools. On the whole, requirements for macro-nutrients for preschool children are roughly about 70% of the requirements of primary schoolchildren. Those of adolescents at the lower secondary level are about 40% higher than those of primary school-age children. It is recommended to use the same commodities (but different ration sizes) for the different age groups whenever the school feeding program targets pre-primary, primary, or lower secondary schools.

Table 2 below presents estimates of recommended energy, protein, and fat intakes for purposes of planning food rations for children and adolescents. Although RNI are age- and sex-specific, for purposes of ration planning for school feeding, wider age ranges are generally used than those of RNI (FAO/WHO/UNU, 2004) and the proposed age groups are: pre-primary: 3 to <6 years; primary: 6 to <12 years, and in some instances lower secondary, 12 to ≤16 years. In all instances, an even distribution by age and sex within each age group is assumed (16). In view of this arbitrary assumption and classification, the term “estimated recommended nutrient intakes (ERNI)” is used in our discussion so as to differentiate from the recommended nutrient intakes (RNI).

**Table 2:** **Estimates of daily macronutrient requirements for children and adolescents**.

Age groups/education level	Age	Daily energy requirements		Estimates of daily RNI		
				Energy	Protein	Fat
	
		Boys	Girls	Boys and girls	Boys and girls (10–15% of energy)	Boys and girls (15–30% of energy)

	(years)	(Kcal)	(Kcal)	(Kcal)	(g)	(g)
Pre-primary/ECD	3–4	1252	1156			
	4–5	1360	1241	1300	33–49	22–43
	5–6	1467	1330			
	Average for 3–6 years	1360	1240			
Primary	6–7	1573	1428			
	7–8	1692	1554			
	8–9	1830	1698			
	9–10	1978	1854	1850	46–69	35–62
	10–11	2150	2006			
	11–12	2341	2149			
	Average for 6–12 years	1930	1780			
Lower secondary	12–13	2548	2276			
	13–14	2770	2379			
	14–15	2990	2449	2600	65–98	44–88
	15–16	3178	2491			
	Average for 12–16 years	2870	2400			

*Source: FAO/WHO/UNU, 2004 (16)*.

*Micronutrients* comprise vitamins and minerals that help to regulate growth, activity, and development, functioning of the immune and reproductive systems, and are needed by the body in minute amounts.

The age grouping used by FAO, UNICEF, UNU, and WHO for nutritional requirements does not coincide with that of UNESCO as regards level of schooling in the education system, particularly for adolescents. Recommended intakes for iron are disaggregated by sex for the subgroups: 11–14 and 15–17. In this case, the 11–14 years sub-group coincides roughly with “early adolescence,” although this differs from UNFPA age grouping of 10–15 years. Recommended iron intakes are much higher for menstruating adolescents (for example, in the case low iron bioavailability, the recommended intake is 32.7 mg/day as compared with 14.0 mg/day for non-menstruating adolescents.) In the case of adolescent girls, the figures retained in the table below are those of non-menstruating adolescents based on a review of various studies, which report average age at menarche (menstruation) to range from 12.5 years in high income countries to 15 and above in poorer countries [WHO (17)]. This figure needs to be verified as there is an indication that age at menarche is progressively decreasing in high-income countries. For iron, the proposed estimate is based on very low iron bioavailability (i.e., 5%). The figure in brackets refers to situations of low iron bioavailability (i.e., 10%). The table below focuses on three micronutrients of great concern in developing countries particularly for adolescent girls and children, namely iron, iodine and vitamin A (Table 3).

**Table 3:** **Recommended micronutrient intakes**.

Age groups (years)	Recommended safe level of intake				Estimates of daily RNI for planning daily rations		
	Iron based on bioavailability mg/day^a^		Iodine μg/day^b^	Vitamin A μg RE/day^c^	Iron mg	Iodine μ*g*	Vitamin A μg RE
		
	Low	Very low					
Pre-primary							
1–3	5.8	11.6	90	400	12 (6)	90	450
4–6	6.3	12.6	90	450			
Primary							
7–10	8.9	18.8	120	500	17.8 (9)	120	500
Lower secondary							
10–18							
Females: 11–14	14	28	150	600	29 (15)	150	600
Males: 11–14	14.6	29.2					

*Age groups*.

^a^*Iron: “Recommended Iron Intake (mean + 2 SD) for diets of different bioavailability” based on UNICEF/UNU/WHO (2001) (33)*.

^b^*Iodine: “Daily Iodine Requirement” based on WHO (2001b) Assessment of the iodine deficiency disorders and monitoring their elimination. Geneva, World Health Organization (31)*.

^c^*Vitamin A: “Recommended Safe Intake” based on FAO/WHO (2001a) Human Vitamin and Mineral Requirements, Report of a joint FAO/WHO Expert Consultation Bangkok, Thailand. (32)*

*Source: FAO/WHO/UNU, 2001 (33)*.

## Country Comparisons

This section examines in detail the school meal provision in five high-income countries (England, France, USA, Italy, and Finland), four middle-income countries (Brazil, Ghana, India, and South Africa), and three low-income countries (Kenya, Mali, and Rwanda).

### High income countries

In *England*, the School Meals Review Panel, appointed by the Government, published its report titled “*Turning the Tables*” (18), which led to the implementation of new food standards regarding food sold or served in schools. The Department for Education and Skills (DfES) established the School Food Trust (SFT) in 2005 and aims “*to promote the education and health of children and young people by improving the quality of food supplied and consumed in schools*” (19). Various funding mechanisms are in place to aid the SFT (20). Nutrient-based food standards came into effect on September 2008 (18). Catering is provided either by the local authority or schools organized their own catering service via private catering services or through an in-house service (21). Local authorities (LAs) either offer an in-house catering service or use a centrally procured private contractor. The average cost of a school meal is $2.58 in primary schools and $2.72 in secondary schools (in 2006–2007) (22). Free school meals (FSM) are available to children from families as per assessment criteria by HM Revenue and Customs (23). A canteen style service is in place in almost all schools with some pupils bringing packed lunches from home. There has been much emphasis on making the dining environment more appealing (24). The SF framework for England is shown in Table 4 and the nutritional guidelines are shown in Table 5.

**Table 4:** **School meal provision frame work table for high-income countries**.

(High income)	England	France	Italy	Finland	USA
Aims and objectives	“Promoting education and health of children and young people by improving the quality of food supplied and consumed in schools” (19)	To ensure that schoolchildren receive essential and high-quality nutrients; current school lunches found to be often high in fat and protein and low in dairy products, fruits, and vegetables (19)	Great emphasis on organic food and sustainability; promotion of Italian farming practices and the Italian diet and food culture (19)	To ensure students in schools and sixth form colleges receive high quality nutritious food; to support the learning of manners and Finnish customs (19)	Improving the health and well-being of the schoolchildren (19)
Policy	The School Food Trust (SFT) was set up by the DfES in 2005 to achieve above-mentioned aim	Ministry of National Education and Minister for Research have set out non-compulsory nutrient and food-based guidelines	The Finance Law 488 ensures that regional and organic sourced foods are promoted	All students in schools and sixth form colleges are entitled to a free meal (19). The meal is required to fulfill one third of the pupil’s RDA	National School Lunch Act passed to achieve above-mentioned aim (29)
Implementation and delivery	Catering is provided by the LA or through private catering service. Las either offer an in-house catering service or a centrally procured private contractor (21) Meals are usually served canteen style and some pupils bring packed lunches from home	Local councils in charge of providing meals. An increase in contracting meals out to private caterers (26) Majority of schools operate a canteen style service. Vending machines are banned in schools (28). If schools do not serve breakfast, guidelines suggest, serve food, preferably a diary product, at least 2 h prior to lunch time (60).	The Italian government invests on ingredients and the school meal service (36). Local authorities purchase food-stuff for meal preparation. Young schoolchildren (aged 2–14) sit at round tables, table cloths, crockery and silverware, and they are served a three course meal with teachers often joining them (36).	Each municipality is responsible for organizing the meals (40). Meals are provided either by the municipality council or a private catering company (19) Cafeteria style service Packed lunches are not allowed (19)	At the federal level, the USDA administers the National School Breakfast Programme and the National School Lunch Programme, whereas at the local level, state education agencies operate the programs (29) Majority operate a canteen style service

*This table compares the aims and objectives, policy and implementation, and delivery of service between high-income countries*.

**Table 5:** **Nutrition guidelines comparison between England, France, USA, Italy, Finland, and Brazil**.

	WHO RDA (10–14 years) (12)	England (61)	France (61)	USA (61)	Italy	Finland	Brazil (62)
Daily ration, g/person/day	–	–	–	–	–	–	–
Energy, Kcal	2210	663 (30%)	884 (40%)	995 (45%)	–	–	660 (30%)
Protein, g	50	15 (30%)	7.5 (15%)	10 (20%)	–	–	20 (40%)
Fat, g	42.1	15 (35%)	15 (35%)	13 (30%)	–	–	16.3 (39%)
Calcium, mg	600	210 (35%)	150 (25%)	180 (30%)	–	–	390 (65%)
Iron, mg	24	8 (35%)	12 (50%)	8 (35%)	–	–	3.2 (13%)
Iodine, μg	140	–	–	–	–	–	–
Vitamin A, μg RE	550	193 (35%)	–	220 (40%)	–	–	210 (38%)
Thiamine, mg	0.90	–	–	–	–	–	–
Riboflavin, mg	1.50	–	–	–	–	–	–
Niacin, mg	14.6	–	–	–	–	–	–
Vitamin C, mg	25	9 (35%)	–	11 (45%)	–	–	18 (72%)
Approximate cost per daily meal	–	$2.58 (41)	$5.54–$7.12 (41)	$1.55 (41)	$4.68 (41)	$2.63 (41)	$0.15 (43)

France aims to ensure that schoolchildren receive essential and high-quality nutrients. Funding for school meals in France is subsidized by approximately 50% by the Ministry of education and the remainder paid by parents, the amount being determined according to their level of employment (25). Local councils are in charge of providing the meals and they are increasingly contracting the meals to private caterers (26). The cost of a school meal varies across France from around $5.54 to $7.12 per child (19). Approximately 50% of schoolchildren eat a school lunch (27). The majority of French schools operate a canteen-style service. School meals are mostly three or four courses. Vending machines were banned in schools in September 2005 (28). The SF framework for France is shown in Table 4 and the nutritional guidelines are shown in Table 5.

In the United States of America (USA), the National School Lunch Act was passed with the aim of improving the health and well-being of the schoolchildren. At the federal level, the United States Department of Agriculture (USDA) administers the National School Breakfast Program and the National School Lunch Programme and at the local level, state education agencies operate the programs (29). It was reported by USDA in 2003 that the program’s cost £7.1 billion (25). The Fresh Fruits and Vegetables Program operate in four states and three tribal organizations (30). School districts use the lowest cost bid approach in order to reduce costs. Reducing costs are deemed as necessary due to stringent federal reimbursement processes. This has led to many questions being raised about the quality of food served (34). The average cost of a canteen lunch is $1.55, (25) with subsidies and free school meals available to families with low-income (35). Majority of the meals are served canteen style. The SF framework for the USA is shown in Table 4 and the nutritional guidelines are shown in Table 5.

In *Italy*, there is great emphasis on organic food and sustainability. The Finance Law 488 ensures that regional and organic sourced foods are promoted, along with Italian farming practices and the Italian food culture. The Government invests on ingredients and the school meal service (36). Due to the promotion of organic foods, almost 60% of LAs purchase these for meal preparation and non-organic food has to be certified under specified regulations (36). GM foods are not permitted. An average school meal costs $4.68 (37). In families which have more than one child attending school, a 20% discount is offered on total cost of the school meal (38). The majority of schoolchildren use a school lunch. The dining experience is considered very important in Italy and much emphasis is placed on Italian food culture and healthy eating. Young schoolchildren (aged 2–14) sit at round tables covered by table cloths and silverware and they are served a three-course meal, with teachers often joining them (36). Meals are provided free for the poorest families, with discounts for low-income families (34). The SF framework for Italy is shown in Table 4. We could not find any nutritional guidelines for Italy.

In *Finland*, since 1983, all students in schools and sixth form colleges are entitled to a free meal (19). This meal is required to fulfill one-third of the pupil’s daily food requirements. This is funded by the Ministry of Social Affairs and Health (39), and each municipality is responsible for organizing the meals and receives an approximate 70% subsidy of the costs from the Government (40). Meals are provided either by the municipality council or a private catering company (19). The cost of a free meal per school child is on average $2.63. The children serve and return the food trays themselves and this reduces labor costs (41). The dining environment is well-furnished and there is great emphasis on the learning of table manners and Finnish customs (19). Packed lunches are not allowed. It is required that a meal must contain a main course, salad, drink, bread, and margarine (19). The SF framework for Finland is shown in Table 4. We could not find any nutritional guidelines for Finland.

### Middle income countries

In *Ghana*, school feeding has existed since 1958, mainly through the Catholic Relief Service (CRS) and the WFP with the main aims of tackling poverty and improving the nutritional status of communities (42). SFPs in Ghana aim to increase enrollment and attendance (42). Food used in the SFP menus of CRS and WFP have historically been imported US food surpluses. However, since 2005, WFP has started to purchase corn, salt, and palm oil locally (42). In 2004, Ghana developed its own national SF program, and as a result of this, 405,000 children receive daily school meals (42). The Ghanaian SFP aims to provide all primary and kindergarten schoolchildren in the poorest areas with a daily nutritious hot meal prepared using local produce. The SF framework for Ghana is shown in Table 6.

**Table 6:** **School meal provision framework table for middle-income countries**.

	Ghana	Brazil	India	South Africa
Aims and objectives	To tackle poverty and improve nutritional status of communities (42) To increase enrollment and attendance (42)	To reduce the number of malnourished children and improve the rates of school enrollment (43) To address the levels of overweight and obesity (43)	To improve the nutritional status of schoolchildren and improve enrollment and retention (44)	To contribute to the quality of teaching and learning through the provision of a nutritious meal to learners (63)
Policy	Provide a nutritious hot meal daily prepared using local produce to all primary and kindergarten schoolchildren in the poorest areas (42)	The Zero Hunger Project (Fome Zero) and Bolsa Família conditional cash transfer program address food security as social policy (43)	Part of policy of the Department of School Education and Literacy and the Ministry of Human Resources Development through the National Steering and Monitoring Committee (44)	Policy and guidelines formulated by the Department of Education. The Conditional Grant Framework (CGF) spells out the conditions for financing, the targeting criteria, and the meal composition (63)
Implementation and Delivery	Administered at the national regional and district levels. District Implementation Committee (DIC) procures food items and runs program.(42)	The national SFP implemented through the School Feeding Committee, which each municipality or state government is required by law to create (43)	The responsibility for cooking the mid-day meal and its supply is normally delegated to a group or organization such as a local women’s or mother’s self-help group, a local youth club, or a voluntary organization (44)	Department of Education, in consultation with the Department of Health, prepares menu options. Provinces select menus based on social acceptance, availability, and cost. The central government pays service providers for the food procurement (63)

*This table compares the aims and objectives, policy and implementation, and delivery of service between middle-income countries*.

The Ghanaian SFP is administered at the national level through a secretariat, accountable to a range of Government Ministries, which formulates policies and establishes institutional structures. Policy and practice is filtered down to the regional and district levels. The regional government coordinates and monitors the SFP and the local government implements the program at the district level through the District Implementation Committee (DIC). It is the responsibility of the DIC to procure food items for the SFP and ensure the running of the program. At the school level, the School Implementation Committee (SIC) sets the menu, employs the cooks and organizes the cooking, and provides the food. The cost of a lunchtime meal per child per day is approximately $0.32 (42). The menu composition and nutritional content of menus vary across Ghana and change by time of year. Menu compositions are shown in Table 7. Detailed menu compositions can be found in Supplementary Material.

**Table 7:** **Comparison of nutrient composition in school feeding menus of Ghana, India, Kenya, Mali, and Rwanda**.

	WHO RDA (10–14 years) (12)	Ghana (42)	India (44)	Kenya (46)	Mali (47)	Rwanda (48)
Daily ration, g/person/day	–	225	178	198	190	141
Energy, Kcal	2210	664 (30%)	680 (31%)	706 (32%)	731 (33%)	537 (24%)
Protein, g	50	16.3 (33%)	30.9 (62%)	24.8 (50%)	17.9 (36%)	14.5 (29%)
Fat, g	42.1	11.1 (26%)	15.7 (37%)	11.5 (27%)	11.1 (26%)	10.1 (24%)
Calcium, mg	600	22 (4%)	158 (26%)	42 (7%)	30 (5%)	153 (25%)
Iron, mg	24	3.7 (16%)	11.4 (47%)	5.8 (24%)	3.9 (16%)	7.8 (32%)
Iodine, μg	140	3 (2%)	183 (131%)	181 (129%)	1 (0%)	180 (129%)
Vitamin A, μg RE	550	375 (68%)	51 (9%)	275 (50%)	104 (19%)	213 (39%)
Thiamine, mg	0.90	0.19 (21%)	0.63 (70%)	0.86 (95%)	0.36 (40%)	0.98 (109%)
Riboflavin, mg	1.50	0.10 (7%)	0.52 (34%)	0.38 (25%)	0.11 (7%)	0.53 (35%)
Niacin, mg	14.6	10.4 (71%)	12.2 (83%)	4.5 (31%)	9.2 (63%)	7.4 (50%)
Vitamin C, mg	25	4 (18%)	4 (17%)	1 (3%)	1 (2%)	0 (0%)
Approximate cost per daily meal	–	$0.32 (42)	–	$0.19 (46)	$0.59 (47)	$0.48 (48)

In *Brazil*, food security is at the center of social policy through the Zero Hunger Project (*Fome Zero*) (43). Part of this project is the *Bolsa Família* program, which gives an amount of money to children from low-income households, and in return, the children are, at the very least, expected to attend school and complete primary level education. A sum of $7.41 per child per month is given to a family with an income less that $59 per capita. For families with incomes of less than $30 per capita, an additional $25 is given. The *Bolsa Família* Program aids over 30 million poor people and is considered as one of the largest conditional cash transfer schemes in the world. Brazil has placed its SFP in its food security policy framework. The SFP aims to reduce the number of malnourished children and improve the rates of school enrollment. Recently, there has been much debate on the nutritional content of the food provided in schools since nearly 40% of the Brazilian population are considered overweight and 5% considered obese (43). The SF framework for Brazil is shown in Table 6.

The SFP is implemented through the School Feeding Committee, which each municipality or state government is required by law to create. Financial transfers are carried out automatically (thus reducing paperwork and other costs) from National Fund for Development of Education to the local governments via 10 installments per year. Public schools receive $0.09 per student and indigenous schools receive $0.17 per student (43). The local governments are required to spend 70% of this money on basic food materials and there is an emphasis on purchasing from local producers to stimulated local economies. The approximate cost of a meal is $0.15 (43). Nutritional guidelines are shown in Table 5.

In *India*, the SFP is known as the mid-day meals (MDM) program (44). It aims to improve the nutritional status of schoolchildren and improve enrollment and retention. In 2009, The Right of Children to Free and Compulsory Education Act was passed, which made it a part of the Constitution that every child has a right to full-time elementary education in a formal school of satisfactory and equitable quality. In 1995, the National Programme of Nutritional Support to Primary Education (NP-NSPE) was launched. The SF framework for India is shown in Table 6.

The MDM program is running at the national level by the Department of School Education and Literacy and the Ministry of Human Resources Development through the National Steering and Monitoring Committee (NSMC) which disseminates policy and guidelines, among various other responsibilities, to the state level (44). There are further Steering and Monitoring Committees (SMCs) at the state and district and these committee oversee and ensure the implementation of the program. At the local level, the responsibility for cooking the mid-day meal and its supply is normally delegated to an organization such as a local women’s self-help group, a local youth club, or a voluntary organization. Menu compositions are shown in Table 7. Detailed menu compositions can be found in Supplementary Material. We could not find the daily cost per mid-day meal per child in India.

In *South Africa*, the school feeding project, known as the National School Nutrition Programme (NSNP), was started in 1994 by President Nelson Mandela as a project of the Reconstruction and Development Programme (RDP) and it targets the poorest areas (Bastia, 2007) (49).

Initially, the aim of the NSNP was stated as “to contribute to the improvement of education quality by enhancing primary pupils’ learning capacity, school attendance, and punctuality, and contribute to general health development by alleviating hunger. Educating pupils on nutrition and also improving nutritional status through micro-nutrition supplementation. Parasite eradication was indicated to develop the nutrition component of the general education curriculum” (64). This lead to some confusion on whether the NSNP was a feeding program, a nutritional intervention, or whether its main objective was to improve educational attainment (64). Therefore, in 2004, the NSNP decided to focus on hunger alleviation instead of its nutritional objectives, as providing a nutritious meal was deemed as too expensive and difficult to monitor and evaluate. The SF framework for South Africa is shown in Table 6.

The Department of Education is responsible for the running of the NSNP, which is financed through a central budget with no reliance on international food donations. Policy and guidelines are formulated here and disseminated via the national coordinator to individual provinces to be implemented.

The Department of Education, with consultation from the Department of Health, prepares the menus, of which there are 22 options for each province to select from. The provinces select the menus based on social acceptance, availability, and cost (45). The traditional South African diet is reflected in the menus with the inclusion of ingredients such as samp (a maize-based meal) and beans.

The central government pays service providers for the food procurement. The NSNP menus are only accessible for the children who are included in the program with other schoolchildren required to bring their own food to school, as food is not allowed to be sold or taken away from the school premises. The cost of providing a lunchtime meal per child per day is approximately $0.32 (Bastia, 2007) (49).

### Low income countries

In *Kenya*, school feeding has been in place for many years and is usually actively supported by parents (46). The aim of the SFP is to increase school enrollment, attendance, and retention, and increase the overall literacy attainment of the country. It mainly targets the Arid and Semi-Arid Lands (ASAL). The Government of Kenya (GoK) started a HGSF program in selected schools around the country in July 2009 (46). The aim of HGSF program was to further build upon the benefits of school feeding by stimulating local economies. The program operates through funds paid by the Government directly to the School Management Committees (SMCs) to purchase cereals, pulses, and oils. SMCs are also in charge of school buildings and the repair and upkeep of school property along with other responsibilities. There have been many challenges in ensuring that the food purchased is from local smallholder farmers due to the fact that Kenya has very little arable land. In 2008, 1.2 million children benefited from the SFP. The SF framework for Kenya is shown in Table 8.

**Table 8:** **School meal provision frame work table for low-income countries**.

(Low income)	Kenya	Mali	Rwanda
Aims and objectives	To increase school enrollment, attendance, and retention, and it mainly targets the Arid and Semi-Arid Lands (ASAL) (46). It aims to increase the overall literacy attainment of the country (46)	To increase enrollment and retention of primary school students. This is an important objective as around 20% of children in this demographic do not attend school (47)	To increase access to education in the short-term and quality of education in the medium-term (48)
Policy	Policy focus on building upon the benefits of school feeding by stimulating local economies (46)	The Ministry of Education implements the SFP as part of its policy (47)	Currently, the SFP is running by a partnership with the WFP. Currently, there are no detailed plans for a SFP in any GoR documents which address education reform or economic development (48)
Implementation and delivery	The program operates through funds paid by the Government directly to the School Management Committees (SMCs) to purchase foodstuff. (46)	The GoM aims to implement it through a decentralized structure with service delivery through school and canteen management committees which will be overseen by the representative from the MoE (47)	The MoE wants individual schools to organize food procurement from local smallholder farmers and parents to provide certain foods to make up the school ration menu and pay for school fees and labor costs for the SFP (48)

*This table compares the aims and objectives, policy and implementation, and delivery of service between low-income countries*.

The MoE is in charge of implementing the HGSF (the WFP is gradually handing over its program to the HGSF) and it has various coordinators at the national, regional, district, and local levels. The MoE has in place a program that builds upon the schools experience in financial matters. Schools already purchase textbooks and other supplies from local businesses and these processes are ensured for transparency through monitoring and evaluation, further enhanced by requiring three signatures to withdraw any funds. The GoK has not taken into account the food cost variation across the country; rather, school budgets are allocated per child based on average costs based on national surveys. This may negatively impact HGSF provision in areas of high food cost.

The HGSF is implemented in the ASALs. These are areas of food deficit and 60–70% of food supplies are in fact imported from outside these areas. These regions have very little water, a small number of farmers, and of these, many focus purely on livestock. Agricultural production by local smallholder farmers is constrained by high production costs. Limited or no storage capacity means more products are prone to waste, reduces entry into markets and other alternatives, and causes farmers to sell surplus stock quickly to bidders who may exploit this urgency.

The Kenyan MoE has not specified a menu or ration composition of its own; rather, it has adopted the WFP’s daily hot lunch ration. As part of the HGSF, cereals, pulses, and oil are purchased from local smallholder farmers (46). Firewood and salt are required to be produced by the parents, along with water and salaries for the cooks (46). If a household is unable to contribute these, then the schools arrange alternative methods of participation with the family (46). Menu compositions are shown in Table 7. Detailed menu compositions can be found in Supplementary Material. The cost of providing a lunchtime meal per child per day is approximately $0.19 (46).

In *Mali*, the primary aim of the SFP conducted by the MoE of the Government of Mali (GoM) is to increase enrollment and retention of primary school students (47). This is an important objective as around 20% of children in this demographic do not attend school (47). The MoE aims for 100% enrollment of primary age schoolchildren by the year 2012. The SF framework for Mali is shown in Table 8.

Mali’s SFP aims to cover about 9,000 basic education or primary schools in the country. The GoM aims to implement it through a decentralized structure with various management committees overseeing the service delivery. Mali has much variation in food supply, access, and availability across the country and from year to year. Sourcing foods for the HGSF program is constrained by variability in the crop yields from 1 year to another, along with low levels of productivity, lack of essential agricultural technology, and the limitation in the diversity of crops, which depends heavily on the amount of rainfall.

The MoE SF policy proposes a partnership between the parents and SMCs. The program relies on developing income generating activities (IGAs) that will raise funds for the upkeep of the program (47). Parents are asked to donate staples (rice, millet, sorghum) and condiments (peanuts, vegetables, seasonings) to help prepare the school meal (47). Menu compositions are shown in Table 7. Detailed menu compositions can be found in Supplementary Material. The cost of providing a lunchtime meal per child per day is approximately $0.59 (47).

In *Rwanda*, the MoE of the Government of Rwanda (GoR) aims to increase access to education in the short-term and quality of education in the medium-term (48). According to the GoR, there is 98% enrollment and 90% attendance rates in schools – it has one of the highest rates of primary school enrollment in sub-Saharan Africa (48). There has been a 70% increase in enrollment after the removal of school fees (48). However, according to the GoR, this has caused a lack of classrooms, teachers, and head-teachers to cater for the increase in students, and it has also put a strain on the education budget. WFP is currently the primary partner of the GoR in the SFP. This is to be gradually developed into a program that is fully government-administered. The program activities will be transferred to government and community entities and WFP support will be phased out, although these plans are yet to be finalized. The MoE wants individual schools to organize food procurement from local smallholder farmers and parents to provide certain foods to make up the school ration menu and pay for school fees and labor costs for the SFP. The MoE believes that if parents are unable to aid the program through these methods, then funds generated from school gardens, animal husbandry, and milk production will be sufficient to sustain the SFP. Currently, there are no detailed plans for a SFP in any GoR documents, which address education reform or economic development. A HGSF program in Rwanda could help students and smallholder farmers greatly by alleviating short-term hunger, increasing long-term food security, increasing income levels, and improving livelihoods, especially for women. The SF framework for Rwanda is shown in Table 8. Menu compositions are shown in Table 7. Detailed menu compositions can be found in Supplementary Material. The cost of providing a lunchtime meal per child per day is approximately $0.48 (48).

## Discussion

School feeding in middle and low-income countries have very different objectives and goals compared to the high-income countries. School meal provision in high-income countries is driven by evidence that the foods children consume in schools are very high in fat and lack adequate amounts of essential nutrients (49, 51). SFPs in middle- and low-income countries, in the short term, aim to alleviate hunger, act as a social safety net for low-income households, and increase enrollment of children into schools; (7) and in the longer term, it aims to improve the nutritional status, attendance, cognitive development, and retention of schoolchildren (7).

The increasing prevalence of childhood overweight and obesity (3–6) has been a major policy-determining factor in the drive for healthy school meal provisions in high-income countries (52). School meal provision in high-income countries is trying to focus on modeling healthier eating habits and food choices that will enable schoolchildren to establish positive dietary habits for the future (18, 52). This is vital toward decreasing the increasing burden of non-communicable diseases on the health care systems of countries (17) and increasingly important toward combating the global obesity epidemic (53). There is also an increasing focus on education on healthier lifestyle choices, as studies have shown that the risk of obesity in children is increased by five times when they are unequipped with adequate nutritional knowledge, which is subsequently complemented by unhealthy eating habits and further negatively impacted by low physical activity levels (54).

In middle and low-income countries, policy has been mainly driven by the need to reduce poverty, establish social safety nets for financially vulnerable households, and to increase and enhance the educational attainment of its population – specifically the primary schoolchildren. Middle and low-income countries are increasingly incorporating local produce into the SF menus by implementing HGSF programs with the aim of stimulating local markets and economies.

### Implementation and delivery

In high-income countries, there is no uniformity in the school meal provision, with modes of provision varying from packed lunches to canteen-style services to children going home for lunch (13, 19, 21, 25, 26, 36, 41). There has been a shift in method of food preparation and delivery, from traditional seated dining with food prepared on-site toward catering delivery services through centrally procured private contractors, vending machines, and school cafeterias (26, 28). In high, middle, and low-income countries, the programs are planned at the national level with local authorities/councils and municipalities responsible for organizing and administering the programs (19, 21, 26, 29, 36, 40). In middle and low-income countries, food is increasingly procured from local farmers and prepared on-site by staff employed by the school, whose salaries the parents of students are responsible for. In some cases, the parents take the responsibility of preparing the meal.

### Nutrition guidelines

We were only able to find nutritional guidelines for England, France, USA, and Brazil. For Ghana, India, Kenya, Mali, and Rwanda, there are no legislated or advised nutritional guidelines; so, we calculated nutritional content from menus specified in the literature found for each country. An important point to note for all countries is that there is no literature verifying the implementation of these guidelines and menus. This is something that requires further research.

Details of comparisons across countries by nutrient types are presented in the following sections.

### Energy

Energy is the product of cellular respiration required for the functioning of the human body. In high-income countries, the focus is on ensuring that schoolchildren do not consume too much in proportion to their energy expenditure, which will lead to obesity. The high-income countries in this study, and Brazil, recommend that the menu should provide 30–45% energy requirements of the RDA (see Table 5). In the remaining middle- and low-income countries, menu compositions indicate that menus contain approximately 30% of energy requirements of the RDA (see Table 7). It is however important to realize that, despite the apparent similarity in energy provision between the country income groups, the energy expenditure is very different between these countries. The school meal provided in middle- and low-income countries tends to be the biggest, or even the only meal, for many schoolchildren on a given day due to poverty. Energy expenditure in schoolchildren in these countries also tends to be higher due to the methods and distance of travel to school, whereas less energy is expended in high-income countries due to better transport and better-placed schools. Schoolchildren in middle- and low-income countries may also have to work before or after school hours in order to support the family and also pay for school fees. It is common for schoolchildren in high-income countries to purchase high-energy foods such as sweets, whereas the vast majority of schoolchildren in middle- and low-income countries are unable to afford these, and therefore do not have complementary methods of energy intake.

### Protein

Proteins are essential for the growth and repair of the human body. Proteins can provide a small amount of energy; however, their main function is building and repairing tissues. Protein-intake guidelines vary in the high-income countries with England and France recommending 30 and 15% of the RDA, respectively (see Table 5). Protein intake in middle- and low-income countries is generally much higher in comparison. Brazilian guidelines recommend 40% of the RDA and menu composition of the rest of the countries ranges from 29% of RDA (Rwanda) to 62% of RDA (India). This increase can be explained by the inclusion of large amounts of cereals (rice mainly) and pulses in comparison to the total of the daily ration. This is a positive factor as it will help schoolchildren in middle- and low-income countries due to the various reasons mentioned previously.

### Fat

Fats have very important roles in the functioning of the human body. They act as an energy store and can be metabolized to produce large amounts of energy. They are vital in the absorption of essential vitamins such as A and D. High levels of fat intake can eventually lead to various diseases such as diabetes and cardiovascular diseases, thus reducing the quality of life of a person. Growing levels of overweight and obesity are a cause for concern in high-income and other countries worldwide. Many studies found that food provided in schools in high-income countries had high levels of fat, and now guidelines in these countries instruct a maximum amount of fat in food: 35% of the RDA in England and France, and 30% in the USA (see Table 5). School meals in the USA were particularly high in fat content, and hence, the lower guideline recommendations. Brazil recommends fat content of approximately 40% of the RDA and this is in attempt to aid schoolchildren to increase their energy storage levels. Menu compositions in the remaining middle- and low-income countries vary from 24% of the RDA in Rwanda to 37% of the RDA in India (see Table 7). Higher levels of fat in menus in the more impoverished regions in these countries will aid schoolchildren to build up their energy stores.

### Iron

Iron is an essential requirement for the human body due to the importance it has in the makeup of the blood and its ability to accept and donate electrons. Lack of adequate levels of iron in the body can lead to fatigue, and eventually, iron deficiency anemia. Too much iron intake can lead to iron overload and hemochromatosis, which can affect organs severely. Nutrition guidelines in the high-income countries instruct iron to make up 35% (England and the USA) and 50% of the RDA (France) (see Table 5). Brazilian guidelines are much lower at 13% of the RDA. More research is required as to why it is set at that low figure. In the remaining middle- and low-income countries, there is much variation (see Table 7) with only India with over 35% of the RDA at 47%.

### Iodine

Iodine is essential to the human body as it is necessary in the production of the vital hormones thyroxin and tri-iodotyronine, which determine the basal metabolic rate of the body. Low levels of iodine intake lead to iodine deficiency, which can cause goiter, cretinism, and other developmental problems. According to a UN report, it is “the primary cause of preventable mental retardation in children and remains a major global public health problem” (55). Nearly three-quarter of a billion school-aged children worldwide are reported to have inadequate iodine intake according to the WHO (56). Adding iodine to salt is a very easy method of preventing this.

We were not able to find any nutritional guidelines for iodine intake in the high-income countries and Brazil. This is most likely due to levels of iodine intake being classified as adequate by the Ministries without need for suggesting guidelines in order to ensure adequate consumption. In the remaining middle- and low-income countries, we found some interesting results. Four countries, India, Kenya, Rwanda, and South Africa, specifically mentioned the inclusion of iodized salt in their menus and these menus provided 131, 129, 129, and 59% of the RDA, respectively. Ghana and Mali did not specifically mention iodized salt in their menus, and therefore was calculated to contain 2 and 0% of the RDA, respectively. Further research is required to better study this element of the menu.

### Vitamin A

Vitamin A is a vital nutrient for the human body and the lack of it can lead to night blindness initially, and eventually total blindness if left unaddressed. Vitamin A deficiency is one of the biggest causes of blindness in developing countries. Guidelines in England and the USA recommend that menus should contain 35 and 40% of the RDA, respectively. France does not have any guidelines for Vitamin A in menus and Brazilian guidelines recommend 38% of the RDA. Menus in Ghana and Kenya have high levels of Vitamin A, 68 and 50% of the RDA, respectively. This is due to the inclusion of palm oil in Ghana and maize in Kenya, both of which are high in Vitamin A content. Rwanda menus have maize meals too; however, the amount served is less hence it delivers 39% of the RDA. Menus in India and South Africa indicate very low Vitamin A content (9 and 2% of the RDA, respectively) and more research needs to be done in order to find out if this is being addressed.

### Costs

The financial aspect of school meals varies. In middle- and low-income countries, school meals are provided free of charge and the government either pays the local authority/municipality in full or subsidizes the cost. In high-income countries, schoolchildren pay for school meals but free school meals are provided to children from households earning below a defined threshold. In Finland, all students in schools and sixth forms are entitled to a free school meal.

There is variation in the cost of providing a school meal for lunch. School meals in high-income countries can be provided for as little as $1.55 per day in certain parts of USA (see Table 5), whereas in France a meal can cost between 5.54 and $7.12. In middle-income countries, it is much cheaper – in Ghana and South Africa the cost of a meal is $0.32 (see Table 7) and $0.15 in Brazil (see Table 5). The cost of a meal in low-income countries in slightly higher, 0.59 and $0.48 in Mali and Rwanda respectively; with the exception of Kenya where a meal costs approximately costs $0.19. The differences in the prices between income groups and within income groups are due to the prices, availability, and procurement methods among many other factors, an analysis of which is beyond the scope of this work.

### Cognitive and psychological impact

Improving the health and nutrition status of school age children can influence learning in school, though the impact pathways are complex. Micronutrients may have a direct cognitive and psychological impact. Though the mechanisms through which iron affects the functioning of the brain and the central nervous system are not yet well understood, there is ample evidence that reduction in iron deficiency improves mental functions across all age groups [Grantham-McGregor and Ani (57); Pollitt (58)]. Iron interventions were found to have a positive impact on infant development scales, IQ tests, and school achievement. Restoration of micronutrient requirements and energy intake can also have an impact on attention and motivation. Energy intake [Pollitt et al. (59)] and iron intake [Grantham-McGregor and Ani (57)] can have an impact on hyperactivity, withdrawal, nervousness, hostile behavior, and happiness. The emotional status of children affects the attention span and has other spill-over effects. A child cannot be physically healthy without also being psychologically secure and a healthy psychosocial environment helps to promote non-violent interaction in both the classroom and the playground. Undoubtedly, nutrition has a positive psychological effect on children with diminished cognitive abilities and sensory impairments. The quality of teaching in class is likely to be affected as teachers may become more motivated and as the quality of students’ performance in class improves.

## Limitations

This analysis is limited by a number of important factors. In countries that did not have any guidelines, we had to compare guidelines with menu compositions. This is not ideal but it was the only accessible method in the scope of this review. Furthermore, the choice of menus in the literature might not be reflective or representative of actual menu compositions at schools as adherences to these menus have not been verified. Alternatively, it would have been possible to obtain a variety of menus and calculated averages. Further research also needs to be conducted in order to verify actual menu compositions being implemented in schools in countries of all income groups. Another point to note is that the analysis involved comparisons between nutritional values to the RDAs of healthy 10- to 14-year-old children. This cannot be used in the case of sick children. Further research on this topic will aid greatly in setting these menus and improving the quality of life of these children.

## Conclusion

In conclusion, the aims of school feeding differ between countries of different income groups. Middle- and low-income countries are increasingly adopting HGSF programs and it would be very useful if guidance would be provided on establishing nutritional guidelines through evidence-based research. New tools have been developed to support meal planning including the Meal Planner for HGSF available online at http://www.hgsf-global.org/en/bank/menu-planner. The challenge would be how to implement it on the ground. The menu composition needs to be tailored to each country’s nutritional needs and the level of the implementation of these guidelines needs to be assessed. Ensuring the provision of healthy foods in schools in all countries is vital to increasing attendance and retention, enhancing nutritional status and cognitive development, combating poverty and obesity, and an important social safety net for low-income households. Collaborative research and subsequent evidence-based policy implementation can greatly enhance and improve the quality of life of millions of children worldwide.

## Conflict of Interest Statement

Salha Hadjivayanis Hamdani currently works at the Partnership for Child Development. Aulo Gelli has previously worked at the Partnership for Child Development. Ruzky Aliyar worked with the Partnership for Child Development for his B.Sc. project.

## Supplementary Material

The Supplementary Material for this article can be found online at http://journal.frontiersin.org/article/10.3389/fpubh.2015.00148
