# Supplementary material for: A Review of Nutritional Guidelines and Menu Compositions for School Feeding Programs in 12 Countries
Source: Front Public Health. 2015 Aug 5;3:148. doi: 10.3389/fpubh.2015.00148 (PMC4524891; doi:10.3389/fpubh.2015.00148)
Supplement: Supplementary file 1 [file Data_Sheet_1.PDF]

Appendix 1 – Detailed nutrient compositions of school feeding menus of Ghana, India, South Africa, Kenya, Mali and Rwanda.

Ration Contents (Ghana)	Daily ration g/person/day(38)	Energy kcal	Protein g	Fat g	Calcium mg	Iron mg	Iodine µg	Vit. A µg RE	Thiamine mg	Riboflavin mg	Niacin mg	Vit. C mg
Rice	150	540	10.5	0.8	14	2.6	0	0	0.15	0.05	8.4	0
Palm Oil	5	44	0	4.9	0	0	0	300	0	0	0	0
Tomato (ripe)	30	5	0.3	0.1	3	0.1	1	75	0.01	0.01	0	4
Onion	10	4	0.1	0	2	0	0	0	0	0	0	1
Beef (moderately fat)	30	71	5.5	5.3	3	1.1	2	0	0.02	0.05	2.0	0
Ration total	225	664	16.3	11.1	22	3.7	3	375	0.19	0.10	10.4	4
WHO RDA (10-14 years) (12)		2210	50	42.1	600	24	140	550	0.90	1.50	14.6	25
% of requirements supplied by ration		30%	33%	26%	4%	16%	2%	68%	21%	7%	71%	18%

Ration Contents (India)	Daily ration g/person/day (40)	Energy kcal	Protein g	Fat g	Calcium mg	Iron mg	Iodine µg	Vit.A µg RE	Thiamine mg	Riboflavin mg	Niacin mg	Vit.C mg
Rice	100	360	7	0.5	9	1.7	0	0	0.10	0.03	5.6	0
Lentils	20	68	5.6	0.2	10	1.8	0	2	0.10	0.05	1.4	1
Beans (soya)	50	208	18.2	10	139	7.9	3	4	0.44	0.44	5.2	3
Vegetable oil	5	44	0	5	0	0	0	45	0	0	0	0
Salt (iodized)	3	0	0	0	0	0	180	0	0	0	0	0

Ration total	178	680	30.9	15.7	158	11.4	183	51	0.63	0.52	12.2	4
WHO RDA (10-14 years) (12)		2210	50	42.1	600	24	140	550	0.90	1.50	14.6	25
% of requirements supplied by ration		31%	62%	37%	26%	47%	131%	9%	70%	34%	83%	17%

Ration Contents (Kenya)	Daily ration g/person/day (41)	Energy kcal	Protein g	Fat g	Calcium mg	Iron mg	Iodine µg	Vit. A µg RE	Thiamine mg	Riboflavin mg	Niacin mg	Vit.C mg
Maize (yellow)	150	525	15	6	20	4.1	0	212	0.58	0.30	3.3	0
Vegetable Oil (fortified)	5	44	0	5	0	0	0	45	0	0	0	0
Legumes (split peas)	40	136	9.8	0.5	22	1.8	1	18	0.28	0.08	1.2	1
Salt (iodized)	3	0	0	0	0	0	180	0	0	0	0	0
Ration total	198	706	24.8	11.5	42	5.8	181	275	0.86	0.38	4.5	1
WHO RDA (10-14 years) (12)		2210	50	42.1	600	24	140	550	0.90	1.50	14.6	25
% of requirements supplied by ration		32%	50%	27%	7%	24%	129 %	50 %	95%	25%	31%	3%

Ration Contents (South Africa)	Daily ration g/person/day (40)	Energy kcal	Protein g	Fat g	Calcium mg	Iron mg	Iodine µg	Vit.A µg RE	Thiamine mg	Riboflavin mg	Niacin mg	Vit.C mg
Rice	40	144	2.8	0.2	4	0.7	0	0	0.04	0.01	2.2	0
Sardines	45	139	9	11.3	180	1.4	10	2	0.02	0.09	5.7	0

Lentils	30	101	8.4	0.3	15	2.7	0	4	0.14	0.08	2	2
Vegetables (cabbage)	10	3	0.2	0.0	5	0.1	0	6	0.02	0	0.1	5
Salt (iodized)	1	0	0	0	0	0	72	0	0	0	0	0
Ration total	126	387	20.4	11.8	204	4.8	82	12	0.22	0.18	10	7
WHO RDA (10-14 years) (12)		2210	50	42.1	600	24	140	550	0.90	1.50	14.6	25
% of requirements supplied by ration		18%	41%	28%	34%	20%	59%	2%	25%	12%	69%	27%

Ration Contents (Mali) (42)	Daily ration g/person/day	Energy kcal	Protein g	Fat g	Calcium mg	Iron mg	Iodine µg	Vit.A µg RE	Thiamine mg	Riboflavin mg	Niacin mg	Vit.C mg
Rice	150	540	10.5	0.8	14	2.6	0	0	0.15	0.05	8.4	0
Pulses (split peas)	30	102	7.4	0.4	17	1.3	1	14	0.21	0.06	0.9	1
Vegetable Oil (fortified)	10	89	0	10	0	0	0	90	0	0	0	0
Ration total	190	731	17.9	11.1	30	3.9	1	104	0.36	0.11	9.2	1
WHO RDA (10-14 years) (12)		2210	50	42.1	600	24	140	550	0.90	1.50	14.6	25
% of requirements supplied by ration		33%	36%	26%	5%	16%	0%	19%	40%	7%	63%	2%

Ration Contents (Rwanda) (43)	Daily ration g/person/day	Energy kcal	Protein g	Fat g	Calcium mg	Iron mg	Iodine µg	Vit.A µg RE	Thiamine mg	Riboflavin mg	Niacin mg	Vit.C mg

Maize meal	100	366	8.5	1.7	110	5.3	0	141	0.83	0.46	5.5	0
Beans (dried)	30	101	6	0.4	43	2.5	0	0	0.15	0.07	1.9	0
Vegetable Oil	8	71	0	8	0	0	0	72	0	0	0	0
Salt (iodized)	3	0	0	0	0	0	180	0	0	0	0	0
Ration total	141	537	14.5	10.1	153	7.8	180	213	0.98	0.53	7.4	0
WHO RDA (10-14 years) (12)		2210	50	42.1	600	24	140	550	0.90	1.50	14.6	25
% of requirements supplied by ration		24%	29%	24%	25%	32%	129 %	39%	109%	35%	50%	0%
